# Summary version of the Standards, Options and Recommendations for palliative or terminal nutrition in adults with progressive cancer (2001)

**DOI:** 10.1038/sj.bjc.6601092

**Published:** 2003-08-15

**Authors:** P Bachmann, C Marti-Massoud, M P Blanc-Vincent, J C Desport, V Colomb, L Dieu, D Kere, J C Melchior, G Nitenberg, B Raynard, P Roux-Bournay, S Schneider, P Senesse

**Affiliations:** 1 Centre Léon Bérard, Lyon, France; 2 Centre François Baclesse; Caen, France; 3 FNCLCC, Paris, France; 4 CHU Dupuytren, Limoges, France; 5 Hôpital Necker, Paris, France; 6 Centre Val d'Aurelle, Montpellier, France; 7 Hôpital Raymond Poincaré, Garches, France; 8 Institut Gustave Roussy, Villejuif, France; 9 Hôpital de I'Archet, Nice, France

**Keywords:** nutritional support, palliative care, practice guideline

The majority of patients with advanced cancer develop malnutrition. This malnutrition has an important impact on quality of life, performance status and immune status. It can be responsible for increased morbidity, particularly infectious complications and thus mortality. In five to more than 20% of patients with cancer, death can be directly related to cachexia in the terminal phase (Inagaki *et al*, 1974; Bozzetti, 1995).

## OBJECTIVES

The objective is to define recommendations for the management of nutrition in adult patients with progressive cancer in the terminal phase.

The main questions addressed are:

- What are the different choices for management (oral feeding and artificial nutrition)?
- What are the possible options for feeding considering the clinical status and the preferences of the patient and their family?
- What are the expected benefits and what criteria should be used for follow-up and decision-making?

The aim of these recommendations is to describe the modalities for palliative nutritional management and artificial nutrition in the palliative phase. The aim of such management is to conserve or restore the best possible quality of life and to control any symptoms that are a source of discomfort or distress. The primary objective cannot be to increase survival at any cost, or solely to improve the nutritional status of the patient. The adverse effects caused by nutritional interventions, particularly artificial nutrition, are sometimes responsible for a deterioration in the quality of life and thus can have a detrimental effect on the real objective of palliative care. Decisions on whether or not to initiate, continue or interrupt active nutritional management are particularly difficult. Not supplying or stopping nutrition is often interpreted by the patient, their family and/or the carers as an abandonment. Food is strongly associated with the image of a potential source of life and of energy.

## METHODOLOGY

The general methodology used has already been described (Fervers *et al*, 2001). For this specific SOR, a multidisciplinary working group was set up by the French National Federation of Cancer Centres (Fédération Nationale des Centres de Lutte Contre le Concer – FNCLCC) to review the best available evidence on palliative or terminal nutrition in adults with progressive cancer.

Medline® was searched using a specific strategy for the period 1991 to April 2001. Web sites specialised in nutrition and palliative care were also searched. The contents pages of the journals: *Supportive Care in Cancer* and *European Journal of Palliative Care* were screened from 1996 to 2000 (5 years). In addition, the reference lists of the articles selected were analysed, and the members of the working group provided references from their personal sources.

Following the selection and critical appraisal of the articles, the working group produced a document with the proposed ‘Standards’, ‘Options’ and ‘Recommendations’ (SORs) for palliative and terminal nutrition in adults with progressive cancer.

When all the members of the working group agree, based on the best available evidence, that a procedure or intervention is beneficial, inappropriate or harmful, it is classified as a ‘Standard’, and when the majority agree, it is classified as an ‘Option’ (Table 1

**Table 1:** Definition of Standards, Options and Recommendations

Standards	Procedures or treatments that are considered to be of benefit, inappropriate or harmful by unanimous decision, based on the best available evidence
	
Options	Procedures or treatments that are considered to be of benefit, inappropriate or harmful by a majority, based on the best available evidence
	
Recommendations	Additional information to enable the available options to be ranked using explicit criteria (e.g. survival, toxicity) with an indication of the level of evidence

). In the SORs, there can be several ‘Options’ for a given clinical situation. ‘Recommendations’ provide additional information that enable the available options to be ranked using explicit criteria (e.g. survival, toxicity) with an indication of the level of evidence. These recommendations thus help clinicians to select an appropriate option. Thus, clinicians can make choices for the management of patients using this information and taking into consideration local circumstances, skills, equipment, resources and/or patient preferences. The adaptation of the SOR to the local situation is allowable if the reason for the choice is sufficiently transparent and this is crucial for successful implementation. Inclusion of patients in clinical trials is an appropriate form of patient management in oncology and is recommended frequently within the SORs, particularly in situations where only weak evidence exists to support a procedure or an intervention.

The type of evidence underlying any ‘Standard’, ‘Option’ or ‘Recommendation’ is indicated using a classification developed by the FNCLCC based on previously published methods. The level of evidence depends not only on the type and quality of the studies reviewed, but also on the concordance of the results (Table 2

**Table 2:** Definition of level of evidence

*Level A*
There exists a high-standard meta-analysis or several high-quality randomised clinical trials that give consistent results

*Level B*
There exist good quality evidence from randomised trials (B1) or prospective or retrospective studies (B2). The results are consistent when considered together

*Level C*
The methodology of the available studies is weak or their results are not consistent when considered together

*Level D*
Either the scientific data do not exist or there is only a series of cases

*Expert agreement*
The data do not exist for the method concerned, but the experts are unanimous in their judgement

). When no clear scientific evidence exists, judgement is made according to the professional experience and consensus of the expert group (‘expert agreement’), and this is then validated by the peer-review process.

The document was then peer-reviewed by independent experts, and their comments were integrated in the final version. The French summary version was based on the full version that has been published (Bachmann *et al*, 2001) and both are available on the FNCLCC web site (http://www.fnclcc.fr). The document will be updated when new scientific evidence becomes available or when there is new expert agreement.

### Definition of palliative care

Palliative care and its organisation should be defined in a consensual manner and controlled by regulations (standard). Nutritional management is defined as a supportive treatment, and in a palliative setting is part of the global management aimed at maintaining or restoring ‘well-being’ (standard). Patients with a life expectancy of less than a month can be considered to be in the terminal phase of their illness (recommendation, expert agreement). Patients with a life expectancy of at least 3 months or more, or with an illness no longer responsive to curative treatment, are considered to be in the palliative phase (recommendation, expert agreement).

### Clinical symptoms and prognostic factors

Gastrointestinal symptoms and nutritional problems are often observed in patients with advanced cancer (standard, level of evidence: B2). Functional scores (Karnofsky index and the WHO performance status) have a good prognostic value in cancer and should be used (standard, level of evidence: B2). Anorexia is a poor prognostic factor in patients with advanced cancer (standard, level of evidence: B2). A Karnofsky score of 50% or lower, or a performance status higher than 2, is associated with a short life expectancy in patients with advanced cancer (recommendation, level of evidence: C). Dyspnoea is indicative of a poor short-term (weeks) prognosis (recommendation, level of evidence: C). The prognostic value of certain biological factors and quality of life scores should be assessed in future prospective studies (recommendation, expert agreement).

### Organisation of management

In France, patients with advanced stage, progressive cancers are cared for in hospital wards and specific palliative care units or at home (standard). Wherever the patient is cared for, the management should be multidisciplinary and the strategy should be discussed with all the different actors involved (standard). The patients and/or their families should be offered appropriate support and whenever possible, their preferences should be respected (standard). Locally written procedures should be available for nutritional management (recommendation, expert agreement).

### Oral feeding

Dietary advice may help to increase feeding and to improve the management of symptoms that interfere with feeding. Specific diets (e.g. low-salt diet) should be stopped or made less strict in order to allow for patients' food preferences (standard). Standard oral supplementation will increase the nutritional uptake in patients with cancer undergoing active treatment (recommendation, level of evidence: B2). Increasing the oral supplementation of eicosapentaenoic acid (EPA) may improve the nutritional status of patients with cachexia secondary to pancreatic cancer (recommendation, level of evidence: B2).

### Symptomatic treatments

Medical treatment of symptoms is necessary in palliative management (standard). Most of these treatments (apart from the appetite stimulants) have not been evaluated in randomised controlled trials in palliative nutritional management (standard). Megesterol acetate, medroxyprogesterone acetate and corticosteroids have an appetite stimulating effect (recommendation, level of evidence: B1) and they may improve the quality of life for patients in the palliative stage (recommendation). Patients with bowel obstruction may benefit from a by-pass procedure, if they suffer from distressing vomiting that cannot be controlled by medical treatment and/or if their life expectancy is prolonged (>3 months) (recommendation, level of evidence: C).

### Pharmaco-nutritional treatment of cachexia

Fish oil derivatives (EPA) may slow the rate of progression of the cachexia (recommendation, level of evidence: B1) these should be evaluated in future studies (recommendation).

### Enteral nutrition

Enteral nutrition in a palliative setting can slow down nutritional deprivation, avoid dehydration and improve the quality of life in patients with head and neck cancer (standard, level of evidence: C). The optimal modalities for delivery, administration and follow-up are the same as those for enteral nutrition when given for other indications (standard, expert agreement). In the terminal or palliative stage, any complications and discomfort resulting from enteral nutrition should be considered. In the event of any change of treatment, the reasons for the change should be discussed with the patients and their families and their preferences taken into consideration (standard, expert agreement). Gastrostomy in terminal-stage patients is associated with a risk of complications that can be contrary to the objectives of palliative care. It is not recommended in this situation (recommendation, level of evidence: C).

### Parenteral nutrition

Parenteral nutrition in the palliative setting can slow down nutritional deprivation, avoid dehydration and improve the quality of life in patients with a malignant bowel obstruction and/or other causes of food intolerance (standard, level of evidence: C). The modalities for delivery, administration and follow-up are the same as those for parenteral nutrition when used for other indications (standard, expert agreement). The benefits expected from parenteral nutrition should be reassessed at regular intervals or each time that a complication related to the technique or the illness occurs (recommendation, expert agreement). There is no justification for parenteral nutrition in patients with a Karnofsky index of 50% or less, or with a performance status score higher than 2 (recommendation, expert agreement).

### Hydration

Dehydration in the terminal phase is often neither painful nor uncomfortable (standard, level of evidence: C). If hydration is administered to control symptoms, the least invasive route should be chosen, for example, the subcutaneous route, if intravenous access is not available (recommendation, expert agreement). Mouth washing is an important component of the management (recommendation, expert agreement). Symptoms can usually be controlled by the subcutaneous administration of 0.5–1.0 l of 0.9% saline solution per 24 h (recommendation, level of evidence: C).

### Therapeutic indications

Routine palliative artificial nutrition is not justified in patients in the terminal phase of their disease since they often do not feel hungry or thirsty, and the benefits have not been demonstrated (standard, expert agreement). The objective of artificial nutrition in a palliative situation is to improve the quality of life (recommendation, expert agreement). This goal may be achieved using artificial nutrition in patients who are unable to eat or to absorb nutrients for a prolonged period of time, and in some of these patients increased survival has been observed (recommendation, level of evidence: C). Artificial nutrition should not be started if the patient's life expectancy is less than 3 months and/or there is any severe, permanent functional deficit (Karnofsky index of 50% or less, or a performance status higher than 2) (recommendation, expert agreement).

### Treatment evaluation

The evaluation of the quality of the nutritional management in patients with cancer in the palliative setting should include assessment of functional scores, quality of life and the patient's (or their family's) satisfaction (standard, expert agreement). Measurement of the nutritional status and the complication rate cannot be used to justify decisions about artificial nutrition, but can be used to assess its quality (standard, expert agreement). Clinical trials using appropriate, validated methodology should be performed to evaluate this management (recommendation, expert agreement).

## Appendix

### Reviewers

P Beau (Hôpital Jean Bernard, La Milétrie, Poitiers, France), RJ Bensadoun (Centre Antoine Lacassagne, Nice, France), JL Bornet (Hôpital de Rangueil, Toulouse, France), D Breilh (Hôpital Haut-Lévêque, Pessac, France), B Burucoa (CHU Pellegrin, Bordeaux, France), J Chalencon (Centre Léon Bérard, Lyon, France), C Chambrier (Hôpital Edouard Herriot, Lyon, France), J Cosnes (Hôpital Rothschild, Paris, France), P Crenn (Hôpital Lariboisière, Paris, France), M DiPalma (Institut Gustave Roussy, Villejuif, France), D Elias (Institut Gustave Roussy, Villejuif, France), H Fernandez (Hôpital Pasteur, Nice, France), M Filbet (Hôpital Val d'Azergues, Alix, France), E Fondrinier (Centre Paul Papin, Angers, France), V Garabige (Institut Gustave Roussy, Villejuif, France), A Gauthier (Polyclinique de Gentilly, Nancy, France), M Hasselmann (Hôpital Hautepierre, Strasbourg, France), J Labrosse (Le Puy en Velay, France), C Lacroix (Centre Val d'Aurelle, Montpellier, France), Y Lallemand (Centre Léon Bérard, Lyon, France), M Magnet (Soin et santé, Caluire, France), J Meuric (Institut Curie, Paris, France), P Morice (Institut Gustave Roussy, Villejuif, France), A Mortureux (Institut Bergonié, Bordeaux, France), F Pinguet (Centre Val d'Aurelle, Montpellier, France), MA Piquet (Centre Hospitaller, Caen, France), JF Ramée (Nantes, France), P Rebattu (Centre Léon Bérard, Lyon, France), C Roberge (Hôpital Bon Sauveur, Caen, France), M Schneider (Centre Antoine Lacassagne, Nice, France), M Simon (Centre Alexis Vautrin, Vandoeuvre lès Nancy, Nancy, France), A Van Gossum (Hôpital Erasme, Bruxelles, Belgium), MP Vasson (Faculté de Pharmacie, Clermond-Ferrand, France), and M Yakoun (Clinique Saint Jean, Montpellier, France).
